# Cardio-cerebral infarction: a narrative review of pathophysiology, treatment challenges, and prognostic implications

**DOI:** 10.3389/fcvm.2025.1507665

**Published:** 2025-03-25

**Authors:** Weiwei Gao, Lingfeng Yu, Jingjing She, Junxuan Sun, Shouyue Jin, Jingjing Fang, Xingyu Chen, Renjing Zhu

**Affiliations:** ^1^ Department of Neurology, Zhongshan Hospital of Xiamen University, School of Medicine, Xiamen University, National Advanced Center for Stroke, Xiamen, China; ^2^ Department of Emergency, Zhongshan Hospital of Xiamen University, School of Medicine, Xiamen University, Xiamen, China; ^3^ Department of Cardiology, West China Xiamen Hospital of Sichuan University, Xiamen, China

**Keywords:** cardio-cerebral infarction, acute myocardial infarction, acute ischemic stroke, narrative review, pathophysiology, treatment strategies, prognosis

## Abstract

Cardio-cerebral infarction (CCI) is a rare clinical syndrome characterized by the simultaneous or sequential occurrence of acute myocardial infarction (AMI) and acute ischemic stroke (AIS). Despite its complex pathogenesis and more severe prognosis compared to isolated AMI or AIS, no consensus has been established regarding its definition, classification, epidemiology, treatment protocols, or prognostic management. Current research is largely confined to case reports or small case series, and there are no unified diagnostic or treatment guidelines, nor any expert consensus. Consequently, clinicians often rely on single-disease guidelines for AMI or AIS, or personal experience, when managing CCI cases. This approach complicates treatment decisions and may result in missed opportunities for optimal interventions, thereby adversely affecting long-term patient outcomes. This narrative review aimed to systematically summarize the definition, classification, epidemiological features, pathogenesis and therapeutic strategies, and prognostic aspects of CCI while thoroughly examining the progress and limitations of existing studies to guide future research and clinical practice. By offering a detailed analysis of reperfusion strategies, antiplatelet therapy, and anticoagulation in CCI patients, this review highlights the safety and efficacy differences among current treatments and explores methods for optimizing individualized management to improve clinical outcomes. Furthermore, this article aimed to enhance clinicians' understanding of CCI, provide evidence-based recommendations for patient care, and outline directions for future research. Ultimately, by refining diagnostic and therapeutic strategies, we aimed to reduce CCI-related mortality and improve long-term prognoses for affected patients.

## 1 Introduction

Acute myocardial infarction (AMI) and acute ischemic stroke (AIS) are leading causes of mortality and disability worldwide, posing significant challenges to public health (1). According to the Global Burden of Disease study 2022 (2), the global prevalence of ischemic heart disease was approximately 315 million, with 9.23 million deaths. As a major subtype of ischemic heart disease, AMI substantially impacts patient survival and prognosis. Concurrently, AIS ranks as the second leading cause of death and the third leading cause of disability globally, with 8.66 million prevalent cases and approximately 3.54 million deaths attributed to AIS in 2022. These conditions not only severely impact patients' quality of life but also impose significant burdens on healthcare systems and socioeconomic resources worldwide.

In 2010, Omar et al. introduced the concept of cardio-cerebral infarction (CCI), defined it as a clinical syndrome characterized by the concurrent or sequential occurrence of AMI and AIS within a short timeframe (3). The pathological mechanisms of CCI are complex, involving various factors such as neurogenic, cardiogenic, and inflammatory processes. As a critical illness with dual cardiac and cerebral injury, CCI is characterized by a narrow therapeutic time window, complicated treatment decisions, and poor prognosis, with significantly higher mortality rates compared to isolated AMI or AIS (4, 5). Consequently, the diagnostic and management challenges of CCI far exceed those posed by single-disease entities. Despite advancements in CCI research, there remain numerous uncertainties regarding its definition, diagnostic criteria, and treatment strategies. Furthermore, current studies are predominantly limited to case reports or small case series, and unified diagnostic guidelines or expert consensus on treatment have yet to be established. As a result, clinicians often rely on disease-specific guidelines for either AMI or AIS, or their own clinical judgment, when managing CCI cases.

Given the high-risk nature and complexity of CCI, a comprehensive understanding of its pathogenesis, clinical characteristics, and prognosis is crucial for developing optimized diagnostic and therapeutic strategies. This narrative review aimed to summarize the current knowledge on the definition, classification, epidemiological characteristics, pathophysiological mechanisms, diagnostic approaches, therapeutic interventions, and prognostic factors associated with CCI. By conducting an in-depth analysis of the available literature, this review seeks to enhance the understanding of CCI, offer evidence-based recommendations for clinical management, highlight limitations in current research, and provide guidance for future research directions. Strengthening clinicians' knowledge of CCI and offering an evidence-based framework for decision-making is critical for optimizing diagnostic and treatment approaches, ultimately reducing CCI-related mortality and improving long-term patient outcomes.

## 2 Methods

We conducted a systematic search of English-language databases, including Web of Science, MEDLINE, BIOSIS Previews, and KCI-Korean Journal Database. We used the search term “Cardio-Cerebral Infarction” and included articles published from database inception until December 31, 2024. The initial search yielded 118 papers. After excluding conference papers (*n* = 12), reviews (*n* = 1), abstracts (*n* = 29), theses (*n* = 4), editorials (*n* = 1), letters (*n* = 1), and patents (*n* = 1), we preliminarily screened 94 papers. Two researchers independently conducted the initial screening based on titles and abstracts, with discrepancies resolved through team discussion. Ultimately, 25 articles were deemed relevant to the research topic. Additionally, we manually reviewed the reference lists and similar articles of the retrieved papers to ensure comprehensiveness. After removing duplicate records, two researchers independently screened the articles based on titles and abstracts, with discrepancies resolved through team discussion.

## 3 Definition

In 2010, Omar et al. (3) first reported a case involving a 48-year-old male who developed a large-area AIS within 1 h following an acute inferior wall and right ventricular transmural AMI. They hypothesized that the sequential occurrence of these two conditions was not coincidental and subsequently introduced the concept of CCI. Since then, an increasing number of CCI cases have been documented and studied. Currently, CCI is mainly classified into two types: synchronous and metachronous (6). Synchronous CCI refers to the simultaneous occurrence of AMI and AIS within a short time frame. However, there is no unified definition of “short time frame” at present. Kijpaisalratana et al. (7) defined synchronous CCI as the occurrence of AIS within 12 h after AMI or the occurrence of AMI within 4.5 h after AIS. De Castillo et al. narrowed the time window to 6 h (8), while the latest research expanded the time window to 24 h (5). Metachronous CCI refers to the sequential occurrence of AMI and AIS, with time intervals ranging from days to 3 months (5, 9–11).

Although synchronous CCI has a relatively clear definition, it still faces numerous challenges in clinical practice. First, the clinical manifestations of AMI and AIS may overlap or mask each other, potentially leading to delayed diagnosis. Second, some patients may experience asymptomatic or “silent” myocardial infarction or cerebral infarction, increasing the difficulty of accurately determining the time of onset. To address this issue, Gao et al. (12) suggested that patients diagnosed with both AMI and AIS at admission, but with unclear sequence and time interval, could be classified as having synchronous CCI. This perspective fully considers the objective difficulties in accurately determining the time of onset in clinical practice, particularly for patients with insidious AMI symptoms. Future studies should validate the definition of synchronous CCI in larger samples and further explore rational diagnostic criteria for metachronous CCI to establish a unified diagnostic consensus and guide clinical practice.

## 4 Epidemiology

Currently, there is no consensus on the incidence of CCI, with considerable differences in results among studies (Table 1). Ho et al. (5) conducted a retrospective cohort study of 120,531 patients with AMI and AIS from the Singapore National Stroke and AMI Registry. They found that 0.5% of patients met the diagnostic criteria for synchronous CCI (AMI and AIS occurring within 24 h), while 0.8% of patients had metachronous CCI (AMI and AIS occurring sequentially within 1 week). However, a single-center study by Yeo et al. (6) involving 555 AIS patients revealed an incidence of synchronous CCI (AMI and AIS occurring within 24 h) of only 0.009%, significantly lower than other studies. Factors contributing to these discrepancies may include small sample sizes and selection bias. De Castillo et al. (8) included 1,683 AIS patients and 1,983 AMI patients, reporting an overall CCI prevalence of 0.79%. Among these, patients who developed AIS within 12 h after AMI or AMI within 6 h after AIS were classified as having synchronous CCI (0.25%), while those who experienced AIS and AMI sequentially within 72 h were classified as having metachronous CCI (0.55%).

**Table 1:** Summary of major studies reporting the prevalence and outcomes of CCI.

Study	Country	Sample size	Population (%)	Prevalence (%)			Time window
				Total	Synchronous	Metachronous	
Kajermo et al. (15)	Sweden	173,233	AMI (100)	2.1	NA	NA	Total: within 30 days
Yeo et al. (6)	Sweden	555	AIS (100)	0.009	NA	NA	Not mentioned
Chong et al. (66)	Singapore	3,500	AIS (100)	NA	0.29	NA	Synchronous: ≤24 h;
Budaj et al. (13)	GRACE	35,233	AMI (100)	0.33	NA	NA	Total: In-hospital
Ho et al. (5)	Singapore	127,919	AMI (NA); AIS (NA)	1.24	0.49	0.76	Synchronous: ≤24 h; Metachronous: ≤1 week
Gao et al. (12)	China	25,229	AMI (30.10); AIS (69.94)	0.34	NA	NA	CCI: 2 weeks
de Castillo et al. (8)	Philippines	3,666	AMI (54.10); AIS (45.90)	0.79	0.25	0.55	Synchronous: AMI ≤12 h before AIS or AIS ≤6 h before AMI; Metachronous: within 72h
Kawamura et al. (14)	Japan	2,281	AMI treated with PCI (100)	0.83	0.53	0.35	Synchronous: ≤24 h post-PCI; Metachronous: 24 h post-PCI to discharge

AMI, acute myocardial infarction; AIS, acute ischemic stroke; CCI, cardio-cerebral infarction; PCI, percutaneous coronary intervention. Study time ranges: Kajermo et al. (1998–2008); Yeo et al. (2009–2014); Chong et al. (2014–2018); Budaj et al. (1999–2003); Ho et al. (2007–2018); Gao et al. (2014–2024); de Castillo et al. (2017–2020); Kawamura et al. (2001–2005).

Some studies only report the overall incidence of CCI. Gao et al. (12) reviewed 17,645 AIS patients and 7,584 AMI patients, finding that 85 cases (0.34%) developed CCI within 2 weeks. The GRACE study included 35,233 hospitalized AMI patients, of whom 116 (0.33%) had complicating ischemic stroke during hospitalization (13). Kawamura et al. (14) analyzed complications in 2,281 AMI patients after percutaneous coronary intervention (PCI), reporting that 20 cases (0.88%) developed stroke, with 0.53% occurring within 24 h after the procedure and 0.35% occurring between 24 h post-procedure and discharge. A large cohort study involving 173,233 AMI patients showed an incidence of AIS of 2.1% within 30 days after AMI (15).

The main reason for the variation in CCI incidence rates among different studies is the lack of uniform diagnostic criteria, particularly regarding the sequence and time window definitions for AMI and AIS. Additionally, heterogeneity in study populations is an important factor influencing incidence estimates. Future studies should employ standardized diagnostic criteria in larger sample populations to obtain precise estimates of CCI incidence. Furthermore, there is an urgent need to explore risk factors for CCI, which is crucial for early recognition and prevention. Only by gaining a thorough understanding of the epidemiological characteristics of CCI can clinical practice be better guided and targeted diagnostic and treatment strategies be developed.

## 5 Etiology and pathophysiology

CCI is a clinical syndrome with complex etiologies and diverse mechanisms, involving multiple pathophysiological processes. Based on the available research evidence, the main causes of CCI can be categorized as follows: atherosclerosis, neurogenic factors, cardiogenic factors, inflammatory responses, and certain systemic diseases.

### 5.1 Atherosclerotic factors

Atherosclerosis is the most common shared cause and risk factor for AMI and AIS (16). An analysis of 200 consecutive autopsy reports revealed a significant correlation between the extent of coronary and cerebral artery atherosclerosis, with coronary atherosclerosis developing earlier than cerebral atherosclerosis. Specifically, atherosclerosis is first observed in the left anterior descending artery, while the most severe lesions occur in the basilar artery and middle cerebral artery (17). Gao et al. (12) demonstrated that the risk factor profile of CCI patients is similar to that of patients with common cardiovascular and cerebrovascular diseases, including hypertension, diabetes, dyslipidemia, smoking, and alcohol consumption. This finding aligns with the results of a meta-analysis conducted by Ng et al. (18), which included 44 CCI patients. The meta-analysis revealed that 65.9% of CCI patients were male, with many having a history of long-term smoking (27.3%) and comorbidities such as hypertension (31.8%), diabetes (15.9%), and dyslipidemia (11.4%). These risk factors are common upstream mechanisms that promote the development of atherosclerosis. When a plaque ruptures, the exposed subendothelial tissue initiates platelet activation and the coagulation cascade, leading to thrombus formation and subsequent distal embolization. This process can result in occlusion of the coronary, carotid, or vertebrobasilar arteries, ultimately causing the concurrent or sequential occurrence of AMI and AIS, termed CCI.

### 5.2 Neurogenic factors

The neurogenic pathogenesis of CCI is primarily based on autonomic nervous dysfunction of the “brain-heart axis”. Brain injury may affect the central or peripheral components of the autonomic nervous system, resulting in excessive activation of cardiac sympathetic nerves and/or parasympathetic inhibition, which in turn leads to various cardiovascular complications. Overactivation of the sympathetic system can elevate catecholamine secretion, inducing myocardial ischemia, injury, or even necrosis, and potentially progressing to heart failure. Conversely, parasympathetic inhibition may cause tachycardia, atrial fibrillation, or ventricular arrhythmias. The insular cortex has been shown to play a pivotal role in central autonomic regulation. Stimulation of the right insula can elicit sympathetic excitation, whereas stimulation of the left insula leads to vagal excitation (7). Therefore, AIS involving the left insular region can disrupt the autonomic nervous system's balance, leading to relative sympathetic hyperactivity, which increases the likelihood of arrhythmias, QT prolongation, and myocardial injury, thereby elevating the risk of AMI (7, 19). A prospective study revealed that, compared to patients with non-insular stroke, patients with left insular stroke had a significantly higher risk of adverse cardiac events (RR = 1.75, 95% CI: 1.02–3.00; *P* = 0.05) (19). Additionally, the catecholamine surge following AIS can trigger stress cardiomyopathy, also known as Takotsubo syndrome, which can present as acute myocardial infarction. Stress cardiomyopathy can promote thrombus formation within the cardiac chambers, which may subsequently embolize to both cerebral and coronary arteries, thereby exacerbating the manifestations of AIS and AMI (20).

### 5.3 Cardiogenic factors

The cardiogenic mechanisms underlying CCI primarily involve cardiogenic embolism and circulatory dysfunction following AMI. Studies have shown that patients with AMI are at significantly increased risk of developing AIS, with this risk persisting in the short term (21). A large community-based study reported that the risk of stroke in the first month after AMI increased 44-fold, with this elevated risk lasting up to three years after the acute event (21).

AMI can trigger AIS through various mechanisms (10, 16, 20, 22). Specifically, AMI can cause regional wall motion abnormalities and left ventricular aneurysms, particularly when involving the left ventricular apex and anterior wall, which result in blood flow stasis, thereby increasing the risk of stroke. Under the influence of factors such as low shear stress, inflammation, and a hypercoagulable state, the coagulation cascade is activated, promoting the formation of left ventricular thrombi (10, 16, 20). These thrombi can embolize to intracranial arteries via the systemic circulation, causing AIS (22). Additionally, the heightened secretion of catecholamines following AMI exacerbates platelet aggregation and thrombus formation, further increasing the risk of AIS (10). Loh et al. (23) found that for every 5% reduction in left ventricular ejection fraction after AMI, the risk of stroke rose by 18%.

Atrial fibrillationis another major contributor to both cerebral embolism and coronary embolism and one of the important causes of CCI (24). In patients with comorbidities such as infective endocarditis, cardiac myxoma, left ventricular wall thrombus, or prosthetic valve thrombus, atrial fibrillation can cause thrombus dislodgement, leading to cerebral embolism and coronary embolism, followed by simultaneous or sequential AMI and AIS (16, 25). Additionally, for patients with right heart failure combined with patent foramen ovale, as the right ventricular pressure increases, right ventricular thrombi may flow back into the systemic circulation through the foramen ovale and embolize to cerebral vessels (3).

PCI is also a risk factor for AIS after AMI. A case report described a 64-year-old woman with AMI who developed neurological deficits 2 days after PCI (26). Gao et al. (12) further verified this mechanism. They found that among CCI patients with AMI as the first manifestation (49.41%, *n* = 42), 26 cases (30.59%) were classified as cardioembolism, of which 6 cases were cerebral infarction after PCI, 16 cases were caused by atrial fibrillation, 3 cases were secondary to left ventricular thrombus dislodgement, and 1 case was caused by both atrial fibrillation and left ventricular thrombus.

Moreover, insufficient effective circulating blood volume after AMI can lead to reduced cerebral perfusion, which may also trigger AIS (9). This situation is more common in patients with acute right ventricular infarction but can also occur in cases of left ventricular infarction accompanied by pump failure. After AMI, due to myocardial damage, the ventricular pumping ability decreases sharply, causing severe hypotension, decompensation of blood pressure autoregulation, and a dramatic decrease in cerebral blood flow, thereby inducing AIS. This type of AIS usually occurs in watershed areas and brainstem regions that are highly sensitive to ischemia and hypoxia (3).

### 5.4 Inflammatory factors

Numerous basic studies have confirmed that after stroke, neuroendocrine disorders and sustained intense inflammatory responses are triggered through the brain-heart axis, leading to an injury storm that increases the risk of AMI (27–29). Research in neuroscience has further revealed the neural pathway basis of this process. The neurons of the amygdala and paraventricular nucleus of the hypothalamus are connected to the spleen through direct neural connections, participating in the regulation of immune responses, stress reactions, and inflammatory processes (30). Immune organs such as the spleen are innervated by a rich sympathetic nervous system, with approximately 98% of the splenic nerves composed of noradrenergic nerve fibers. After stroke, these neural pathways are activated, resulting in the massive release of monocytes and neutrophils from the spleen and other immune organs and altering the proliferative state of lymphocytes (31, 32). Furthermore, elevated levels of adrenaline and noradrenaline after stroke can trigger a catecholamine storm, further increasing the levels of inflammatory cytokines in the plasma, activating the hypothalamic-pituitary-adrenal axis, and leading to the secretion of large amounts of endogenous glucocorticoids (33–35). These factors work together to ultimately cause a series of pathological changes, including lysis, necrosis, apoptosis, and fibrosis of myocardial cells; vascular endothelial swelling; basement membrane disintegration; perivascular edema; subendocardial hemorrhage; decreased mitochondrial function; and impaired myocardial excitation-contraction coupling and contractile function, significantly increasing the risk of CCI (36).

Similarly, AMI can trigger a widespread inflammatory response, resulting in the release of large quantities of inflammatory cytokines, which subsequently activate neutrophils and promote the synthesis of acute-phase reactants (24, 37). This cascade may further destabilize and even rupture atherosclerotic plaques in the cerebral circulation (38). Previous studies have shown that C-reactive protein (CRP) levels rise significantly following myocardial injury (39, 40). CRP exerts potent proinflammatory and procoagulant effects (41), reducing nitric oxide production by endothelial cells while simultaneously increasing the expression of adhesion molecules. It also influences monocyte chemotaxis and foam cell formation within atherosclerotic plaques, exacerbating the vasoreactivity of unstable plaques (42). Moreover, a study found that the incidence of complex and unstable carotid plaques in AMI patients was markedly higher than in those with stable angina (42% vs. 8%) (43). Consequently, systemic vascular inflammation induced by AMI may lead to the rupture of multiple plaques and the formation of extensive thrombi in the aorta and cerebral circulation, significantly increasing the risk of AIS.

### 5.5 Other contributing factors

In addition to the mechanisms previously discussed, certain systemic diseases can also contribute to the development of CCI. These include essential or hereditary thrombocythemia, antiphospholipid antibody syndrome, multiple myeloma, and other hematological disorders, as well as hypercoagulable states, vasculitis, and coronary artery disease induced by electrical injury or malignant neoplasms (21, 44, 45). A study revealed that elevated D-dimer levels in the malignancy-associated hypercoagulable state were closely related to extracellular vesicles (EVs) secreted by tumor cells (46). EVs can activate platelet aggregation and promote the formation of neutrophil extracellular traps through tissue factor-dependent and tissue factor-independent pathways, thereby accelerating the thrombus formation process (47, 48). In this hypercoagulable state, extensive microthrombus formation can lead to the simultaneous or sequential development of AIS and AMI (49). Notably, during the COVID-19 pandemic, there was a significant increase in CCI case reports (50, 51). This may be attributed to endothelial dysfunction and abnormal coagulation caused by SARS-CoV-2 infection, which predisposes patients to a hypercoagulable state, thus increasing the risk of CCI.

Aortic dissection, especially type I aortic dissection, is also a significant cause of CCI. The intimal tear in type I aortic dissection originates in the ascending aorta and extends distally, involving the aortic arch and descending aorta (16). When the dissection propagates proximally to the ostium of the carotid artery or vertebrobasilar artery or distally to involve the ostium of the coronary arteries, it can lead to AIS or AMI, respectively (21).

## 6 Management strategies

### 6.1 Acute reperfusion strategies

**Figure 1:**
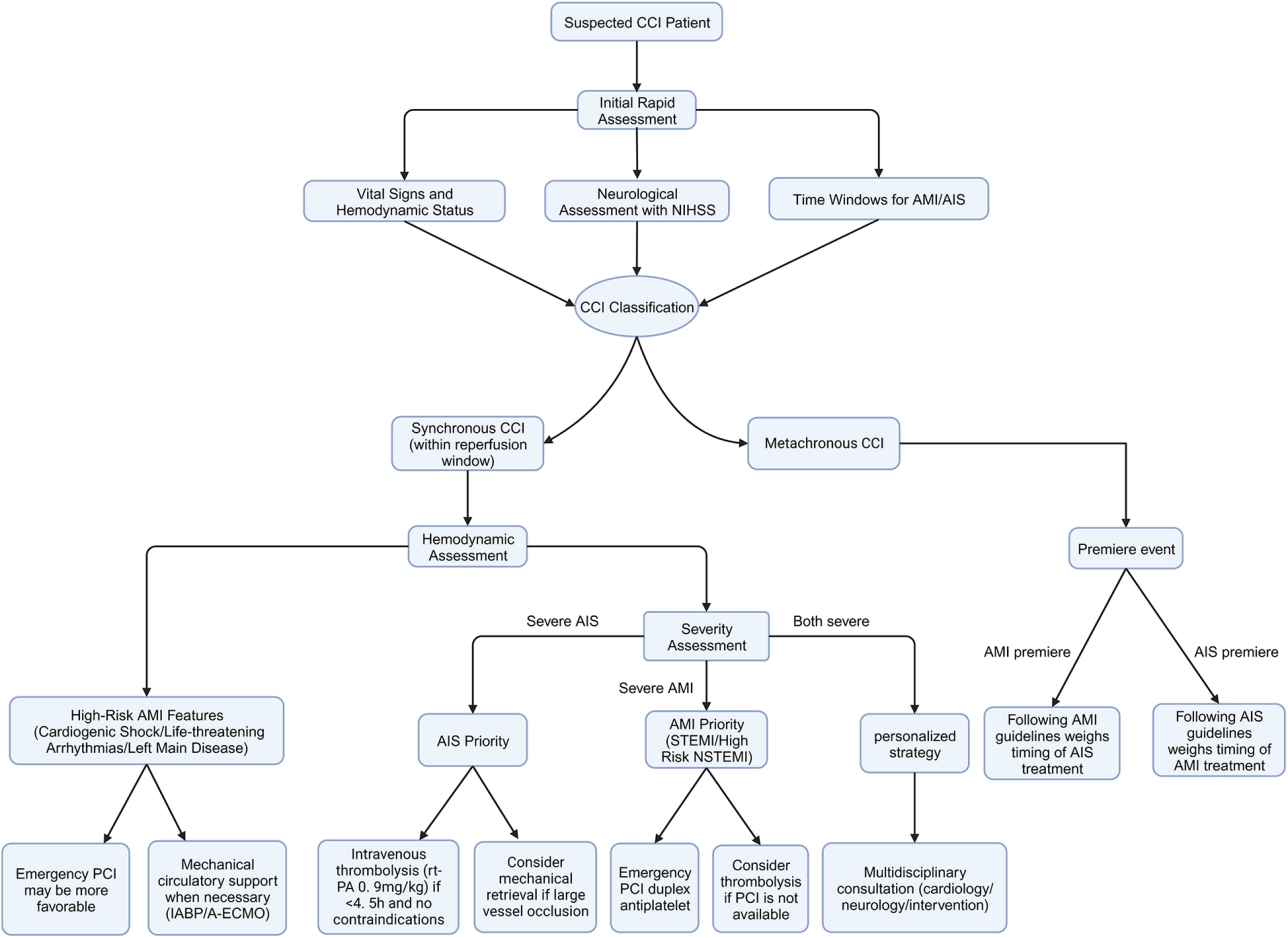
Proposed management strategy for cardio-cerebral infarction. A flowchart illustrating the proposed management strategy for patients with suspected CCI. CCI, cardio-cerebral Infarction; NIHSS, National Institutes of Health Stroke Scale; AMI, acute myocardial infarction; AIS, acute ischemic stroke; STEMI, ST-elevation myocardial infarction; NSTEMI, non-ST-elevation myocardial infarction; PCI, percutaneous coronary intervention.

Rapid restoration of cardiac and cerebral tissue perfusion is the primary principle in the treatment of CCI (Figure 1). The 2019 guidelines of the American Heart Association/American Stroke Association suggest that, for patients with both AIS and AMI, intravenous alteplase at the dose appropriate for AIS should be prioritized, followed by coronary angiography and percutaneous coronary intervention (PCI) as needed (Class IIa recommendation, Level of Evidence C) (52). However, these guidelines do not fully account for the clinical heterogeneity of AMI, such as the type (STEMI/NSTEMI) and severity (presence of life-threatening conditions or complications), and therefore may not be applicable to all CCI patients. In clinical practice, treatment strategies for CCI should be individualized based on the severity, hemodynamic status, and time window for both AMI and AIS onset (7).

Currently, the optimal protocol for intravenous thrombolysis in CCI patients remains uncertain. The recommended dose of rt-PA for AMI (100 mg), which is higher than the dose used for AIS, may increase the risk of symptomatic intracranial hemorrhage (18, 53, 54). Conversely, the standard dose for AIS (0.9 mg/kg, with a maximum of 90 mg) may not be sufficiently effective in treating AMI (5). Additionally, the occurrence of AMI within 3 months is considered a relative contraindication for thrombolysis in AIS patients, while an AIS within the past 6 months is considered an absolute contraindication for thrombolysis in AMI patients (52, 55). These contraindications further complicate the use of intravenous thrombolysis in CCI patients.

Endovascular intervention is another crucial strategy for restoring cardiac and cerebral blood flow. PCI can improve the success rate of myocardial revascularization and reduce adverse cardiac events, with potential benefits even in patients with cardiogenic shock (56). In CCI patients, the intervention strategy must balance the priorities of coronary and cerebrovascular ischemia. For high-risk patients, such as those with cardiac arrest, pulseless ventricular tachycardia/ventricular fibrillation, cardiogenic shock, or left main disease, emergency PCI should be prioritized to maintain cardiac function (7, 57, 58).

Several studies have evaluated the safety and efficacy of PCI in CCI patients. Watanabe et al. (56) reported on 7 CCI patients who underwent percutaneous coronary intervention (PCI). Despite receiving dual antiplatelet therapy before the procedure, 2 patients died, but the remaining 5 did not experience complications such as intracranial hemorrhage. A retrospective study by Schmidbauer et al. (59) indicated that PCI does not increase the risk of intracranial hemorrhage in AIS patients, even during the acute phase of ST-elevation myocardial infarction (STEMI) or in hemodynamically unstable conditions. Mehta et al. (60) analyzed 2,525 patients who received PCI and were simultaneously diagnosed with AIS and STEMI during hospitalization. The results showed that although patients with AIS and concomitant STEMI had a higher mortality rate, PCI treatment was not associated with mortality (OR = 0.93, 95% CI: 0.60–1.43, *P* = 0.7) or an increased risk of intracranial hemorrhage (OR = 1.54, 95% CI: 0.93–2.56, *P* = 0.1). However, this study did not include patients with NSTEMI, and it is unclear whether these patients met the diagnostic criteria for synchronous or metachronous CCI.

For CCI patients with mild manifestations of AMI (such as NSTEMI) or hemodynamically stable but severe neurological symptoms, and whose onset of CCI is within the thrombolytic time window (<4.5 h), intravenous thrombolytic therapy can be administered at the standard dose for AIS first. Subsequently, for patients with large vessel occlusion, bridging mechanical thrombectomy (MT) can be performed (61). Sakuta et al. reported a CCI patient with stable vital signs who first underwent MT to recanalize the occluded intracranial artery and then underwent PCI for the treatment of a coronary artery lesion, resulting in a favorable clinical outcome (57). These cases suggest that, when conditions permit, prioritizing intravenous thrombolysis and MT is a reasonable treatment option, especially for patients with severe neurological impairment, and it may help to further improve cardiovascular symptoms.

In conclusion, for CCI patients, the decision to administer intravenous thrombolysis followed by vascular interventions (PCI/MT) or to prioritize emergency PCI depends on a comprehensive assessment of the severity and urgency of the cardiocerebral ischemic events. An individualized treatment plan should be formulated. Regardless of the chosen strategy, close monitoring and proactive prevention of recurrent thrombi and embolisms are critical to patient management.

### 6.2 Antiplatelet therapy

Antiplatelet therapy is a key component in the management of CCI, and strategies must balance the different clinical needs of AMI and AIS patients. Commonly used antiplatelet agents include aspirin and P2Y12 receptor antagonists, such as clopidogrel and ticagrelor. In AMI patients, oral antiplatelet therapy is necessary regardless of the reperfusion strategy employed, typically starting with a loading dose followed by a transition to a maintenance dose (55). However, the antiplatelet strategy for AIS patients needs to be individualized based on previous treatment. Current guidelines recommend that for AIS patients who are not eligible for intravenous thrombolysis or endovascular thrombectomy and have no contraindications, oral aspirin therapy should be initiated as soon as possible after the onset of symptoms (Class I recommendation, Level of Evidence A). For patients who undergo thrombolytic therapy, antiplatelet agents should be administered 24 h post-thrombolysis (Class I recommendation, Level of Evidence B) (62).

For CCI patients, the use of antiplatelet drugs should be based on a comprehensive consideration of reperfusion strategies, risk-benefit balance, clinical presentation, imaging results, and patient comorbidities to develop individualized treatment plans. A well-chosen antiplatelet regimen can effectively prevent thrombosis recurrence while minimizing the risk of bleeding. Several studies have retrospectively analyzed the use of antiplatelet drugs in CCI patients. Ho et al. (5) included 1,591 CCI patients (625 synchronous and 966 metachronous cases) and found that the proportion of patients receiving antiplatelet therapy was similar between the two groups (80.8% vs. 79.4%), with comparable rates of aspirin (68.0% vs. 67.3%) and P2Y12 receptor antagonist (54.2% vs. 62.1%) use. However, further analysis revealed that patients with AIS following AMI had higher rates of aspirin (76.7% vs. 56.5%) and P2Y12 receptor antagonist (71.1% vs. 51.9%) use compared to those with AMI following AIS. Gao et al. (12) compared the antiplatelet treatment status of 85 CCI patients, categorized into in-hospital death (*n* = 26) and survival (*n* = 59) groups. The results showed that the survival group had a significantly higher rate of dual antiplatelet therapy (DAPT) use (74.58% vs. 42.31%, *P* = 0.014), while the death group had a significantly higher proportion of patients not receiving any antiplatelet therapy (19.23% vs. 6.78%, *P* = 0.014). This difference may be related to the more complex and severe clinical situations faced by patients in the death group (e.g., larger infarct size, higher bleeding risk, or more complications), leading clinicians to adopt a more conservative treatment strategy. While the above studies provided valuable insights into antiplatelet use patterns in CCI, the lack of granular data on specific antiplatelet agents and doses used is a limitation. Future studies should aim to collect and report such details to enable more precise analyses of the impact of different antiplatelet regimens on clinical outcomes in CCI.

### 6.3 Anticoagulation therapy

Anticoagulation therapy plays distinct roles in the management of AIS and AMI, which presents challenges in developing optimal treatment strategies for CCI patients. For most AIS patients, current guidelines do not recommend routine anticoagulation therapy (Class I recommendation, Level of Evidence A). However, for patients with specific indications for anticoagulation, such as atrial fibrillation, anticoagulation therapy is advised 24 h after thrombolysis (Class I recommendation, Level of Evidence B).

AMI patients typically receive anticoagulant therapy in addition to antiplatelet treatment during initial hospitalization and revascularization (63, 64). The 2023 European Society of Cardiology (ESC) guidelines for the management of acute coronary syndromes state that STEMI patients should receive anticoagulant therapy during invasive procedures. However, there is a lack of high-quality evidence regarding the benefit of anticoagulant therapy at early time points in patients undergoing a direct PCI strategy. Furthermore, the guidelines recommend that patients with non-ST-segment elevation acute coronary syndrome (NSTE-ACS) receive non-oral anticoagulant therapy. For NSTE-ACS patients undergoing immediate or early angiography, unfractionated heparin (UFH) is recommended, but enoxaparin should be considered as an alternative to UFH. For NSTE-ACS patients not expected to undergo early angiography, fondaparinux is recommended (64).

For AMI patients who develop left ventricular thrombus, oral anticoagulation therapy is indicated. Direct oral anticoagulants such as apixaban, rivaroxaban, and dabigatran, or vitamin K antagonists (e.g., warfarin/coumadin) are recommended for the treatment of left ventricular thrombus. These anticoagulants help prevent thrombus expansion and embolization to the cerebral circulation, which can cause AIS.

In CCI patients, Gao et al. (12) found that the frequency of anticoagulant therapy was significantly higher in the in-hospital death group compared to the survival group (69.23% vs. 44.07%, *P* = 0.037). De Castillo et al. (8) retrospectively analyzed the treatment strategies of 29 CCI patients, with 66% of patients receiving DAPT combined with anticoagulant therapy, 21% receiving DAPT, and 7% receiving either single antiplatelet therapy or no antiplatelet therapy.

In summary, antiplatelet and anticoagulant therapies are crucial components in the management of CCI patients, requiring the development of individualized regimens based on comprehensive assessments. Clinicians should closely monitor patients' coagulation function and clinical symptoms, promptly adjusting treatment strategies to balance the therapeutic needs of stroke and myocardial infarction while minimizing the risks of bleeding and thrombosis recurrence. Future prospective studies are needed to further explore the optimal antiplatelet and anticoagulant treatment strategies for CCI patients.

## 7 Prognosis and clinical outcomes

AMI and AIS are both major critical clinical emergencies, and their simultaneous occurrence significantly worsens the prognosis compared to either condition occurring in isolation. Several studies have consistently demonstrated that CCI patients face a significantly higher risk of mortality, increased complications, prolonged hospitalization, and greater medical expenses. A meta-analysis by Ng et al. (18) (*n* = 44) revealed the high lethality of CCI, with an overall mortality rate of 22.7%, and 90% of the deaths were related to cardiac complications. Gao et al. (12) further confirmed this finding, reporting an in-hospital mortality rate of 30.59% for CCI patients, with 65.38% of patients dying from cardiac causes. Another larger meta-analysis (4) comprehensively evaluated the short-term and mid-term prognoses of CCI patients and found that the in-hospital mortality rate was as high as 33.3%, with a 3-month mortality rate reaching 49.2%. De Castillo et al. (8) found that the all-cause mortality rate in CCI patients was 45% (33% for synchronous CCI vs. 50% for metachronous CCI), with the majority being cardiovascular deaths (69%).

Ho et al. (5) analyzed the prognosis of 625 synchronous CCI and 966 metachronous CCI patients, finding similar in-hospital all-cause mortality rates between the two groups (35.5% vs. 36.2%). However, compared to patients with isolated AMI or AIS, both synchronous and metachronous CCI patients had significantly increased 30-day mortality risk [synchronous CCI: adjusted hazard ratio (aHR) 2.41, 95% confidence interval (CI) 1.77–3.28; metachronous CCI: aHR 2.80, 95% CI 2.11–3.73; isolated AMI: aHR 2.90, 95% CI 1.87–4.51; isolated AIS: aHR 4.36, 95% CI 3.03–6.27]. Alqahtani et al. (65) conducted a comparative study to investigate the differences in prognosis between CCI patients and those with AIS alone. The results revealed that the in-hospital mortality rate of CCI patients was significantly higher than that of patients with AIS alone (21.4% vs. 7.1%, *P* < 0.0001). Moreover, the incidence of major complications, such as acute kidney injury, hemorrhagic transformation, and gastrointestinal bleeding, was also significantly increased in CCI patients. Additionally, the discharge rate of CCI patients after being cured or improved was significantly lower than that of patients with AIS alone (22.1% vs. 38.4%, *P* < 0.001). From the perspective of medical resource utilization, CCI patients had a significantly longer average length of hospitalization (9 days vs. 6 days, *P* < 0.0001) and higher hospitalization expenses (USD 12,830 vs. USD 9,369, *P* < 0.001) compared to patients with AIS alone. These data collectively reflect the poor prognosis and substantial socioeconomic burden associated with CCI. Overall, compared to patients with isolated AMI or AIS, CCI patients face not only higher short- and mid-term mortality risks but also endure more complications, longer hospital stays, and significantly higher medical expenses (61).

## 8 Conclusion

This review systematically summarized the definition, epidemiological features, pathogenesis and therapeutic strategies, and prognosis of CCI, providing an evidence-based foundation for clinical practice and outlining directions for future research. CCI, characterized by the concurrent or sequential occurrence of AMI and AIS, involves a range of complex pathophysiological mechanisms, including atherosclerosis, neurogenic factors, inflammation, cardiac factors, and certain systemic diseases. Although significant progress has been made in understanding CCI, numerous challenges remain. For instance, classification criteria are not yet standardized, and there is currently no consensus on the optimal diagnostic approach for CCI, given the variability in healthcare settings, resources, and expertise across different regions. Specific treatment guidelines are lacking, treatment strategies remain controversial, and additional high-quality prospective studies are needed to develop approaches for improving long-term outcomes. Establishing standardized diagnostic criteria and protocols for CCI remains an important area for future research.

Furthermore, high-quality clinical research evidence on CCI remains relatively scarce. Given these limitations, there is an urgent need for large-sample, multi-center prospective cohort studies and registries to accurately assess the incidence, risk factor profile, and natural history of CCI. Randomized controlled trials (RCTs), the gold standard for evaluating the efficacy of interventions, should become one of the key research directions in the CCI field. It is imperative to systematically evaluate the impact of different reperfusion strategies, antiplatelet, and anticoagulant regimens on the short- and long-term prognosis of CCI patients through rigorously designed RCTs. However, considering the low incidence of CCI, traditional large-scale RCTs may face numerous practical challenges, such as difficulties in patient enrollment, long study duration, and high costs. Therefore, when designing future RCTs, researchers could consider adopting novel study paradigms, such as sequential trials or basket trials, to improve research efficiency while ensuring study quality. Additionally, developing and validating risk assessment models and personalized diagnostic and treatment decision tools for high-risk CCI populations is of great importance for promoting early recognition and precise management of CCI. Moreover, secondary prevention in CCI patients is another key focus for future research. Optimizing multidisciplinary collaborative pharmacotherapy strategies, strengthening risk factor management, and guiding patients to establish healthy lifestyles will help improve the long-term prognosis of CCI patients.

In conclusion, CCI is a complex clinical syndrome requiring close collaboration among multidisciplinary teams in neurology, cardiology, and critical care. Continued basic and clinical research will help to better understand the pathogenesis of CCI and refine diagnostic and therapeutic strategies, ultimately improving patient outcomes and alleviating the societal burden of the disease. Future studies should prioritize addressing existing knowledge gaps, paving the way for the development of CCI-specific diagnostic and treatment guidelines, and offering more accurate and effective individualized treatment options for high-risk patients.

## References

[1] Virani SS Alonso A Benjamin EJ Bittencourt MS Callaway CW Carson AP Heart disease and stroke statistics-2020 update: a report from the American Heart Association. Circulation. (2020) 141:e139–596. 10.1161/CIR.0000000000000757 31992061

[2] Mensah GA Fuster V Murray CJL Roth GA, Global Burden of Cardiovascular Diseases and Risks Collaborators. Global burden of cardiovascular diseases and risks, 1990–2022. J Am Coll Cardiol. (2023) 19:2350–473. 10.1016/j.jacc.2023.11.007 PMC7615984 38092509

[3] Omar HR Fathy A Rashad R Helal E. Concomitant acute right ventricular infarction and ischemic cerebrovascular stroke; possible explanations. Int Arch Med. (2010) 3:25. 10.1186/1755-7682-3-25 20977759 PMC2974668

[4] Habib M Elhout S. Outcomes of intervention treatment for concurrent cardio- cerebral infarction: a case series and metaanalysis. J Cardiol Cardiovasc Med. (2023) 8:4–11. 10.29328/journal.jccm.1001147

[5] Ho JS Zheng H Tan BY Ho AF Foo D Foo LL Incidence and outcomes of cardiocerebral infarction: a cohort study of 2 national population-based registries. Stroke. (2024) 55:2221–30. 10.1161/STROKEAHA.123.044530 39082144

[6] Yeo LLL Andersson T Yee KW Tan BYQ Paliwal P Gopinathan A Synchronous cardiocerebral infarction in the era of endovascular therapy: which to treat first? J Thromb Thrombolysis. (2017) 44:104–11. 10.1007/s11239-017-1484-2 28220330

[7] Kijpaisalratana N Chutinet A Suwanwela NC. Hyperacute simultaneous cardiocerebral infarction: rescuing the brain or the heart first? Front Neurol. (2017) 8:664. 10.3389/fneur.2017.00664 29270151 PMC5725403

[8] de Castillo LLC Diestro JDB Tuazon CAM Sy MCC Añonuevo JC San Jose MCZ. Cardiocerebral infarction: a single institutional series. J Stroke Cerebrovasc Dis. (2021) 30:105831. 10.1016/j.jstrokecerebrovasdis.2021.105831 33940364

[9] Ibekwe E Kamdar HA Strohm T. Cardio-cerebral infarction in left MCA strokes: a case series and literature review. Neurol Sci. (2022) 43:2413–22. 10.1007/s10072-021-05628-x 34590206 PMC8480750

[10] Boyanpally A Cutting S Furie K. Acute ischemic stroke associated with myocardial infarction: challenges and management. Semin Neurol. (2021) 41:331–9. 10.1055/s-0041-1726333 33851390

[11] Marto JP Kauppila LA Jorge C Calado S Viana-Baptista M Pinho-E-Melo T Intravenous thrombolysis for acute ischemic stroke after recent myocardial infarction: case series and systematic review. Stroke. (2019) 50:2813–8. 10.1161/STROKEAHA.119.025630 31436141

[12] Gao W Yu L Jin S Cai L Fang J Wang X Clinical features and in-hospital mortality predictors of concurrent cardio-cerebral infarction: insights from a dual-center retrospective study. Front Neurol. (2024) 15:1465144. 10.3389/fneur.2024.1465144 39474370 PMC11520769

[13] Budaj A Flasinska K Gore JM Anderson FA Jr Dabbous OH Spencer FA Magnitude of and risk factors for in-hospital and postdischarge stroke in patients with acute coronary syndromes: findings from a global registry of acute coronary events. Circulation. (2005) 111:3242–7. 10.1161/CIRCULATIONAHA.104.512806 15956123

[14] Kawamura A Lombardi DA Tilem ME Gossman DE Piemonte TC Nesto RW. Stroke complicating percutaneous coronary intervention in patients with acute myocardial infarction. Circ J. (2007) 71:1370–5. 10.1253/circj.71.1370 17721013

[15] Kajermo U Ulvenstam A Modica A Jernberg T Mooe T. Incidence, trends, and predictors of ischemic stroke 30 days after an acute myocardial infarction. Stroke. (2014) 45:1324–30. 10.1161/STROKEAHA.113.001963 24692479

[16] Aggarwal G Patlolla SH Aggarwal S Cheungpasitporn W Doshi R Sundaragiri PR Temporal trends, predictors, and outcomes of acute ischemic stroke in acute myocardial infarction in the United States. J Am Heart Assoc. (2021) 10:e017693. 10.1161/JAHA.120.017693 33399018 PMC7955313

[17] Mathur KS Kashyap SK Kumar V. Correlation of the extent and severity of atherosclerosis in the coronary and cerebral arteries. Circulation. (1963) 27:929–34. 10.1161/01.cir.27.5.929 13933662

[18] Ng TP Wong C Leong ELE Tan BY Chan MY Yeo LL Simultaneous cardio-cerebral infarction: a meta-analysis. QJM. (2022) 115:374–80. 10.1093/qjmed/hcab158 34051098

[19] Laowattana S Zeger SL Lima JA Goodman SN Wittstein IS Oppenheimer SM. Left insular stroke is associated with adverse cardiac outcome. Neurology. (2006) 66:477–83. 10.1212/01.wnl.0000202684.29640.60 16505298

[20] Akinseye OA Shahreyar M Heckle MR Khouzam RN. Simultaneous acute cardio-cerebral infarction: is there a consensus for management? Ann Transl Med. (2018) 6:7. 10.21037/atm.2017.11.06 29404353 PMC5787723

[21] Witt BJ Brown RD Jr Jacobsen SJ Weston SA Yawn BP Roger VL. A community-based study of stroke incidence after myocardial infarction. Ann Intern Med. (2005) 143:785–92. 10.7326/0003-4819-143-11-200512060-00006 16330789

[22] Vaitkus PT Barnathan ES. Embolic potential, prevention and management of mural thrombus complicating anterior myocardial infarction: a meta-analysis. J Am Coll Cardiol. (1993) 22:1004–9. 10.1016/0735-1097(93)90409-t 8409034

[23] Loh E Sutton MS Wun CC Rouleau JL Flaker GC Gottlieb SS Ventricular dysfunction and the risk of stroke after myocardial infarction. N Engl J Med. (1997) 336:251–7. 10.1056/NEJM199701233360403 8995087

[24] Wang X Li Q Wang Y Zhao Y Zhou S Luo Z A case report of acute simultaneous cardiocerebral infarction: possible pathophysiology. Ann Palliat Med. (2021) 10:5887–90. 10.21037/apm-21-808 34107694

[25] Takemoto K Nakamura M Atagi K. Concomitant acute pulmonary embolism, myocardial infarction and ischemic stroke due to paradoxical embolism from a patent foramen ovale: a case report. Oxf Med Case Reports. (2021) 2021:omab101. 10.1093/omcr/omab101 34729199 PMC8557459

[26] Kawarada O Yokoi Y. Brain salvage for cardiac cerebral embolism following myocardial infarction. Catheter Cardiovasc Interv. (2010) 75:679–83. 10.1002/ccd.22330 20020521

[27] Shi K Tian DC Li ZG Ducruet AF Lawton MT Shi FD. Global brain inflammation in stroke. Lancet Neurol. (2019) 18:1058–66. 10.1016/S1474-4422(19)30078-X 31296369

[28] Mo Y Sun YY Liu KY. Autophagy and inflammation in ischemic stroke. Neural Regen Res. (2020) 15:1388–96. 10.4103/1673-5374.274331 31997797 PMC7059569

[29] Ishikawa H Tajiri N Vasconcellos J Kaneko Y Mimura O Dezawa M Ischemic stroke brain sends indirect cell death signals to the heart. Stroke. (2013) 44:3175–82. 10.1161/STROKEAHA.113.001714 24008571 PMC3859251

[30] Zhang X Lei B Yuan Y Zhang L Hu L Jin S Brain control of humoral immune responses amenable to behavioural modulation. Nature. (2020) 581:204–8. 10.1038/s41586-020-2235-7 32405000

[31] Zhou Y Zhang Y Cui M Zhang Y Shang X. Prognostic value of the systemic inflammation response index in patients with acute ischemic stroke. Brain Behav. (2022) 12:e2619. 10.1002/brb3.2619 35588444 PMC9226852

[32] Courties G Frodermann V Honold L Zheng Y Herisson F Schloss MJ Glucocorticoids regulate bone marrow B lymphopoiesis after stroke. Circ Res. (2019) 124:1372–85. 10.1161/CIRCRESAHA.118.314518 30782088 PMC6483874

[33] Ding Y DeGracia D Geng X Ding Y. Perspectives on effect of spleen in ischemic stroke. Brain Circ. (2022) 8:117–20. 10.4103/bc.bc_53_22 36267438 PMC9578309

[34] Stephens R Grainger JR Smith CJ Allan SM. Systemic innate myeloid responses to acute ischaemic and haemorrhagic stroke. Semin Immunopathol. (2023) 45:281–94. 10.1007/s00281-022-00968-y 36346451 PMC9641697

[35] Candelario-Jalil E Dijkhuizen RM Magnus T. Neuroinflammation, stroke, blood-brain barrier dysfunction, and imaging modalities. Stroke. (2022) 53:1473–86. 10.1161/STROKEAHA.122.036946 35387495 PMC9038693

[36] Sposato LA Hilz MJ Aspberg S Murthy SB Bahit MC Hsieh CY Post-stroke cardiovascular complications and neurogenic cardiac injury: JACC state-of-the-art review. J Am Coll Cardiol. (2020) 76:2768–85. 10.1016/j.jacc.2020.10.009 33272372

[37] Kassem-Moussa H Mahaffey KW Graffagnino C Tasissa G Sila CA Simes RJ Incidence and characteristics of stroke during 90-day follow-up in patients stabilized after an acute coronary syndrome. Am Heart J. (2004) 148:439–46. 10.1016/j.ahj.2004.01.028 15389230

[38] Neumann FJ Ott I Gawaz M Richardt G Holzapfel H Jochum M Cardiac release of cytokines and inflammatory responses in acute myocardial infarction. Circulation. (1995) 92:748–55. 10.1161/01.cir.92.4.748 7543831

[39] Buffon A Biasucci LM Liuzzo G D'Onofrio G Crea F Maseri A. Widespread coronary inflammation in unstable angina. N Engl J Med. (2002) 347:5–12. 10.1056/NEJMoa012295 12097534

[40] Mauriello A Sangiorgi G Fratoni S Palmieri G Bonanno E Anemona L Diffuse and active inflammation occurs in both vulnerable and stable plaques of the entire coronary tree: a histopathologic study of patients dying of acute myocardial infarction. J Am Coll Cardiol. (2005) 45:1585–93. 10.1016/j.jacc.2005.01.054 15893171

[41] Motro M Barbash GI Hod H Roth A Kaplinsky E Laniado S Incidence of left ventricular thrombi formation after thrombolytic therapy with recombinant tissue plasminogen activator, heparin, and aspirin in patients with acute myocardial infarction. Am Heart J. (1991) 122:23–6. 10.1016/0002-8703(91)90753-5 1905875

[42] Chan AW Bhatt DL Chew DP Reginelli J Schneider JP Topol EJ Relation of inflammation and benefit of statins after percutaneous coronary interventions. Circulation. (2003) 107:1750–6. 10.1161/01.CIR.0000060541.18923.E9 12665489

[43] Lombardo A Biasucci LM Lanza GA Coli S Silvestri P Cianflone D Inflammation as a possible link between coronary and carotid plaque instability. Circulation. (2004) 109:3158–63. 10.1161/01.CIR.0000130786.28008.56 15184282

[44] Casanova M Cornú E Mesa RD. Hyperacute cardio- cerebral infarction: a therapeutic challenge. SN Compr Clin Med. (2021) 3:1804–8. 10.1007/s42399-021-00931-2

[45] Wang L Mao X Zheng J Gu X Yang J. A case of simultaneous acute cardio-cerebral infarction in a woman with essential thrombocythemia. J Int Med Res. (2019) 47:4557–61. 10.1177/0300060519865062 31426696 PMC6753560

[46] Seok JM Kim SG Kim JW Chung CS Kim GM Lee KH Coagulopathy and embolic signal in cancer patients with ischemic stroke. Ann Neurol. (2010) 68:213–9. 10.1002/ana.22050 20695014

[47] Hisada Y Mackman N. Cancer cell-derived tissue factor-positive extracellular vesicles: biomarkers of thrombosis and survival. Curr Opin Hematol. (2019) 26:349–56. 10.1097/MOH.0000000000000521 31261175 PMC6677240

[48] Bang OY Chung JW Lee MJ Kim SJ Cho YH Kim GM Cancer cell-derived extracellular vesicles are associated with coagulopathy causing ischemic stroke via tissue factor-independent way: the OASIS-CANCER study. PLoS One. (2016) 11:e0159170. 10.1371/journal.pone.0159170 27427978 PMC4948859

[49] Gao W Li H Zhang Y Li S Chen X Zhu R. Reperfusion therapy for trousseau syndrome-related cerebral infarction: a case-control analysis of efficacy and prognosis. J Coll Physicians Surg Pak. (2024) 34:910–5. 10.29271/jcpsp.2024.08.910 39113508

[50] Desai R Mondal A Prasad A Vyas A Jain A Rupareliya C Concurrent cardio-cerebral infarctions in COVID-19: a systematic review of published case reports/series. Curr Probl Cardiol. (2023) 48:101814. 10.1016/j.cpcardiol.2023.101814 37209804 PMC10193814

[51] Eskandarani R Sahli S Sawan S Alsaeed A. Simultaneous cardio-cerebral infarction in the coronavirus disease pandemic era: a case series. Medicine (Baltimore). (2021) 100:e24496. 10.1097/MD.0000000000024496 33530272 PMC7850703

[52] Powers WJ Rabinstein AA Ackerson T Adeoye OM Bambakidis NC Becker K Guidelines for the early management of patients with acute ischemic stroke: 2019 update to the 2018 guidelines for the early management of acute ischemic stroke: a guideline for healthcare professionals from the American Heart Association/American stroke association. Stroke. (2019) 50:e344–418. 10.1161/STR.0000000000000211 31662037

[53] Álvarez-Sabín J Maisterra O Santamarina E Kase CS. Factors influencing haemorrhagic transformation in ischaemic stroke. Lancet Neurol. (2013) 12:689–705. 10.1016/S1474-4422(13)70055-3 23726850

[54] Brott TG Haley EC Jr Levy DE Barsan W Broderick J Sheppard GL Urgent therapy for stroke. Part I. Pilot study of tissue plasminogen activator administered within 90 min. Stroke. (1992) 23:632–40. 10.1161/01.str.23.5.632 1579958

[55] Chinese Society of Cardiology of Chinese Medical Association, Editorial Board of Chinese Journal of Cardiology. 2019 Chinese society of cardiology (CSC) guidelines for the diagnosis and management of patients with ST-segment elevation myocardial infarction. Zhonghua Xin Xue Guan Bing Za Zhi. (2019) 47:766–83. 10.3760/cma.j.issn.0253-3758.2019.10.003 31648459

[56] Watanabe T Kobara S Amisaki R Yamamoto K. Primary percutaneous coronary intervention for cardio-cerebral infarction: a case report. Front Cardiovasc Med. (2023) 10:1165735. 10.3389/fcvm.2023.1165735 37583581 PMC10424438

[57] Sakuta K Mukai T Fujii A Makita K Yaguchi H. Endovascular therapy for concurrent cardio-cerebral infarction in a patient with trousseau syndrome. Front Neurol. (2019) 10:965. 10.3389/fneur.2019.00965 31555206 PMC6742686

[58] Bao CH Zhang C Wang XM Pan YB. Concurrent acute myocardial infarction and acute ischemic stroke: case reports and literature review. Front Cardiovasc Med. (2022) 9:1012345. 10.3389/fcvm.2022.1012345 36386323 PMC9663457

[59] Schmidbauer ML Rizas KD Tiedt S Dimitriadis K. Low rate of intracerebral hemorrhage after cardiac catheterization in patients with acute ischemic stroke in a large case series. Clin Neurol Neurosurg. (2020) 198:106159. 10.1016/j.clineuro.2020.106159 32829200

[60] Mehta S Kakouros N Mir T Loree S Qureshi W. Prevalence and outcomes of patients with acute ischemic stroke with concomitant ST-segment-elevation myocardial infarction (results from national inpatient sample 2016–2019). Stroke. (2024) 55(5):1245–53. 10.1161/STROKEAHA.123.044550 38529635

[61] Obaid O Smith HR Brancheau D. Simultaneous acute anterior ST-elevation myocardial infarction and acute ischemic stroke of left middle cerebral artery: a case report. Am J Case Rep. (2019) 20:776–9. 10.12659/AJCR.916114 31154453 PMC6561139

[62] Chinese Society of Neurology, Chinese Stroke Society. Chinese Guidelines for diagnosis and treatment of acute ischemic stroke 2018. Chinese Journal of Neurology. (2018) 51:666–82. 10.3760/cma.j.issn.1006-7876.2018.09.004

[63] Chinese Medical Association, Chinese Medical Journals Publishing House, Chinese Society of General Practice. Guideline for primary care of non-ST-segment elevation acute coronary syndrome (2019). Chin J Gen Pract. (2021) 20:6–13. 10.3760/cma.j.cn114798-20201030-01112

[64] Byrne RA Rossello X Coughlan JJ Barbato E Berry C Chieffo A 2023 ESC guidelines for the management of acute coronary syndromes. Eur Heart J Acute Cardiovasc Care. (2024) 13:55–161. 10.1093/ehjacc/zuad107 37740496

[65] Alqahtani F Aljohani S Tarabishy A Busu T Adcock A Alkhouli M. Incidence and outcomes of myocardial infarction in patients admitted with acute ischemic stroke. Stroke. (2017) 48:2931–8. 10.1161/STROKEAHA.117.018408 29018137

[66] Chong CZ Tan BY Sia CH Khaing T Litt Yeo LL. Simultaneous cardiocerebral infarctions: a five-year retrospective case series reviewing natural history. Singap Med J. (2022) 3:686–90. 10.11622/smedj.2021043 PMC9815165 33866711

